# Bone mass and geometry of the tibia and the radius of master sprinters, middle and long distance runners, race-walkers and sedentary control participants: A pQCT study

**DOI:** 10.1016/j.bone.2009.03.660

**Published:** 2009-07

**Authors:** D.C. Wilks, K. Winwood, S.F. Gilliver, A. Kwiet, M. Chatfield, I. Michaelis, L.W. Sun, J.L. Ferretti, A.J. Sargeant, D. Felsenberg, J. Rittweger

**Affiliations:** a Institute for Biomedical Research into Human Movement and Health, Manchester Metropolitan University, Manchester M1 5GD, UK; b MRC Human Nutrition Research, Cambridge, UK; c Centre for Muscle and Bone Research, Charité – University Medicine Berlin, Free University and Humboldt-University Berlin, Germany; d Center for P-Ca Metabolism Studies, National University of Rosario, Rosario, Argentina; e Faculty of Human Movement Science, Vrije Universiteit, Amsterdam, The Netherlands

**Keywords:** Veteran athletes, Track and field runners, Race-walking, Bone strength, Volumetric bone mineral density, Exercise

## Abstract

Mechanical loading is thought to be a determinant of bone mass and geometry. Both ground reaction forces and tibial strains increase with running speed. This study investigates the hypothesis that surrogates of bone strength in male and female master sprinters, middle and long distance runners and race-walkers vary according to discipline-specific mechanical loading from sedentary controls.

Bone scans were obtained by peripheral Quantitative Computed Tomography (pQCT) from the tibia and from the radius in 106 sprinters, 52 middle distance runners, 93 long distance runners and 49 race-walkers who were competing at master championships, and who were aged between 35 and 94 years. Seventy-five age-matched, sedentary people served as control group.

Most athletes of this study had started to practice their athletic discipline after the age of 20, but the current training regime had typically been maintained for more than a decade. As hypothesised, tibia diaphyseal bone mineral content (vBMC), cortical area and polar moment of resistance were largest in sprinters, followed in descending order by middle and long distance runners, race-walkers and controls. When compared to control people, the differences in these measures were always > 13% in male and > 23% in female sprinters (*p* < 0.001). Similarly, the periosteal circumference in the tibia shaft was larger in male and female sprinters by 4% and 8%, respectively, compared to controls (*p* < 0.001). Epiphyseal group differences were predominantly found for trabecular vBMC in both male and female sprinters, who had 15% and 18% larger values, respectively, than controls (*p* < 0.001). In contrast, a reverse pattern was found for cortical vBMD in the tibia, and only few group differences of lower magnitude were found between athletes and control people for the radius.

In conclusion, tibial bone strength indicators seemed to be related to exercise-specific peak forces, whilst cortical density was inversely related to running distance. These results may be explained in two, non-exclusive ways. Firstly, greater skeletal size may allow larger muscle forces and power to be exerted, and thus bias towards engagement in athletics. Secondly, musculoskeletal forces related to running can induce skeletal adaptation and thus enhance bone strength.

## Introduction

The relevance of mechanical stimuli for bone adaptation has been considered for over hundred years. In 1892 Wolff suggested that bones are structurally adapted to fulfil their mechanical role [1]. Almost a century later, Frost proposed the mechanostat theory, which postulates that continuous adaptation to external forces allows strains, placed on the bone tissue, to be kept within certain limits [2].

Impact forces, particularly from non-typical directions such as those experienced during jumping, tennis or skiing, have been associated with greater bone mass in cross-sectional studies involving young adult athletes [3–6]. Longitudinal intervention studies in children and adolescents [7,8] underline the causative role for such impact forces to enhance bone strength. In addition, studies of exercises that involve large forces without impact, such as weight lifting, have also reported greater bone mineral mass and density along with surrogates of bone strength [5,9,10].

Running disciplines provide the unique opportunity to investigate the association of mechanical loading and bone measures in humans, since external forces, as estimated by ground reaction force measurements, have been suggested to increase as a function of speed [11,12]. Comparisons of ground reaction force and tibial strain measurement studies imply that peak external forces are translated into peak bone strains [11,13]. Accordingly, Nilsson and Westlin [14] have shown that runners have greater areal bone mineral density (aBMD) than control people, and Bennell et al. [15] have demonstrated systematic differences in leg aBMD among sprinters, middle distance and endurance runners, and young control adults. One might therefore expect inter-group variations in bone strength also between runners and race-walkers and sedentary control people.

To the best of our knowledge, however, no studies have investigated differences in tibial characteristics of male and female sprinters, long distance runners, middle distance runners or race-walkers by using peripheral Quantitative Computed Tomography (pQCT) as the measurement technique. Compared to other bone densitometry techniques, for example dual X-ray absorptiometry, pQCT offers the important advantages of assessing volumetric bone mineral density, providing transversal geometric bone characteristics and distinguishing between cortical and trabecular bone [16].

Based on the reports in literature greater running speed is associated with both higher ground reaction forces, but unchanged stride cycle length, and thus also with greater rate of force development. This translates into greater tibial strains and strain rates [11,13]. Accordingly, we expected athletes to have adapted in tibial bone structural characteristics according to their discipline-specific mechanical loading. Therefore, we hypothesised that tibial measures of master sprinters, long and middle distance runners and race-walkers would vary in descending order from the ones of sedentary control participants. In contrast, we hypothesised that the non-weight bearing radius would show no group differences.

## Materials and methods

### Participants

Three hundred track and field master athletes aged between 35 and 94 years and 75 control participants were included in this study (Table 1). Competing runners or race-walkers were recruited by flyers and personal communication at World, European and British Master Athletics Championships between the years 2004 and 2006. Recruitment targeted athletes who ranked highest in previous competitions or who had qualified for semi-finals or finals at the current event. Since most athletes compete in more than one event, they were asked to self-rate their best discipline. Those who considered their best discipline to be either running or race-walking were invited to take part in the study. Measurements in the master athletes were taken during the championships. For the analysis, athletes were classified into four different event categories, depending on their self-rated best discipline: sprinters (100 m, 200 m, 400 m), middle distance runners (800 m, 1500 m), long distance runners (5 km, 10 km, marathon) and race-walkers (5 km, 10 km, 20 km). A comparison of the athletes' best age-graded performance with the self-rated best discipline within a subset of 256 athletes showed 95% concordance and therefore yielded that self rating is a valid indicator of the individual athletic specialisation.

Control participants were members of the local University of the Third Age or employees of Manchester Metropolitan University, UK. They were recruited via flyers, staff emails and personal communication. Measurements took place at Manchester Metropolitan University. The criteria for inclusion of subjects into the control group were that they were mentally active, as evidenced by occupation or educational participation, but they took little physical activity: less than two hours of endurance type exercise per week, no exhaustive and no resistive exercise. Gardening, heavy housework and profession-related physical activities were considered as exercise. The eligibility of the control participants was established by a telephone conversation along with a personal interview and a questionnaire. The questionnaire addressed previous sport participation, general physical activities and physical activities carried out during the past seven days. For all participants, any musculoskeletal disorders known to affect the bones and pregnancy were exclusion criteria. All participants gave their written informed consent before inclusion into the study, which had been approved by the local ethics committee of Manchester Metropolitan University and, in addition, by the local ethics committees in the respective country that the competitions were held in.

### Measurements

#### Participant characteristics

Participants' height and body mass were measured and a health questionnaire was completed to assess medical conditions, medication, injuries, gynaecological status in females, alcohol consumption and smoking behaviour. Additionally, athletes were asked about their competition level and training history, including the starting age, training sessions per week and years of active participation in organised sport, which was club sport but not physical education classes at school.

#### pQCT-scans

Tomographic scans were obtained with two XCT2000 scanners (Stratec Medizintechnik GmbH, Pforzheim, Germany), and image analyses were performed with the integrated software version 5.40 D of one of the scanners. Both scanners were calibrated initially by the manufacturer with the European Forearm Phantom, and their compliance was better than 2% (personal communication J. Willnecker, Stratec). During the study, they underwent daily quality assessment. Scans were obtained at the right lower leg and the right forearm. In the case of a fracture of the right limb within the last 24 months the left side was scanned. The scan locations at the forearm were based on the ulna length measured between the olecranon and the ulnar styloid process. The length of the tibia was assessed as the distance between the palpated medial knee joint cleft and the medial malleolus. Cross-sectional images were obtained after scout viewing the tibio-talar joint and the wrist. Scans were taken at 4% and 60% of the ulna length as well as at 4% and 38% of the tibia length from distal to assess both epiphyseal and diaphyseal bone measures. A voxel size of 0.5 mm in the transverse plane and of 2.4 mm in the longitudinal axis was used. The epiphyseal and diaphyseal bone measures were determined with a segmentation threshold of 180 mg/cm^3^ and 650 mg/cm^3^, respectively.

Names for endpoint variables were chosen according to the Task Force on Standardization of Bone Structure and Density Assessment (http://nomenclature.bb.asbmr.org/) for high-resolution pQCT. The analysed measures at the epiphysis were trabecular volumetric bone mineral content (vBMC.tb) and the trabecular bone mineral density (vBMD.tb) along with the total cross-sectional area (Ar.tot). At the diaphysis, the following measures were analysed: cortical vBMD (vBMD.ct), which was corrected for partial volume effects [17], total vBMC (vBMC.tot) and cortical bone area (Ar.Ct), density weighted polar moment of resistance (RPol) as well as the endocortical and the periosteal circumferences (EnC and PsC, respectively). It should be noted here that RPol in this study is identical to the so-called polar “SSI” that is given by the XCT software. The axial moments of inertia have also been analysed and found to yield almost identical information as RPol, and they are therefore not reported here.

In addition, the circularity of the tibial midshaft cross-section was quantified as: circ = R_Circ / RPol, where R_Circ is the density weighted polar moment of inertia of the ring model of the XCT software. As R_Circ denotes a ring shape around a circular marrow space, R_Circ assumes the least polar moment of resistance for a given vBMC and marrow space size. Hence, the shaft cross-sectional area is perfectly circular when circ = 1 and any deviation from circularity will lead to reductions in circ.

The *in vivo* precision of pQCT measurements of the laboratory is described elsewhere [18,19]. It ranges between 0.2–0.5% for tibial Ar.Tot, Ar.Ct and vBMC.tot and 1.3–1.7% for RPol [18,19].

#### Statistical analysis

Descriptive summary statistics were calculated as means and their standard deviations (SD) or, in the case of figures, ratios of the athletes' and controls' bone measures along with 95% confidence intervals (CI). To test discipline and sex interactions, two-way analyses of variance (ANOVA) were carried out of both absolute and relative values. Interactions were never significant and are therefore not presented. Differences between all athlete groups and the control participants were tested by ANOVA analyses, using simple contrasts. The groups were sprinters, middle and long distance runners, race-walkers and controls. To adjust for the skeletal constitution independent of loading, we included the respective radius measure as covariate in the ANOVA model of the tibia. These analyses revealed similar significances than the crude model and are therefore not presented in detail. Furthermore, there was no consistent evidence that radius and tibia lengths differed between the groups and adjusting for limb length did not affect the results; hence crude analyses are presented throughout this paper. Statistical analysis was performed using SPSS 11 software for Mac OS X (SPSS Inc^®^, Chicago, IL, USA) and STATA 10 (StataCorp, College Station, TX, USA). Significance was assumed if *p* < 0.05.

## Results

### Participants

One hundred and six male and female sprinters, 52 middle distance runners, 93 long distance runners, 49 race-walkers and 75 physically non-active controls participated in the study. Participants' characteristics are given in Table 1. The athletes were aged between 35 and 94 years and the controls between 33 and 89 years. The mean age of the groups ranged between 54 years and 62 years (*p* > 0.05; Table 1). There were no group differences in height, but all athlete groups had lower body mass than control participants (*p* < 0.05). No significant difference was found in age-graded performance among the different athletic groups.

The participation in organised sports during young adulthood is summarised in Table 2. As can be seen from the table, very few control participants practiced in sports when they were young. A large fraction of the master athletes, on the other hand, did participate in organised sports during young adulthood. Their competitions were mostly at local or national, but not at international level, suggesting that the study cohort contains practically no former elite athletes in a strict sense.

Table 3 gives an overview of the types of sport that athletes engaged in before puberty and during young adulthood. Juvenile participation in high or odd impact sports (*e.g.* ball sports) outweighed participation in running and race-walking for all athletic groups, with the exception of sprinters, of whom 27% also practiced sprint running during young adulthood. Overall, however, juvenile participation in running and race-walking was rather low, suggesting that the majority of master athletes have picked up their discipline after the age of 20 years.

As can be seen from Table 4, the athletes were quite persistent in their current training regime, which on average had been maintained for more than a decade in all groups. Finally, about 60% of the females were post-menopausal.

### Tibia pQCT data

**Fig. 1:**
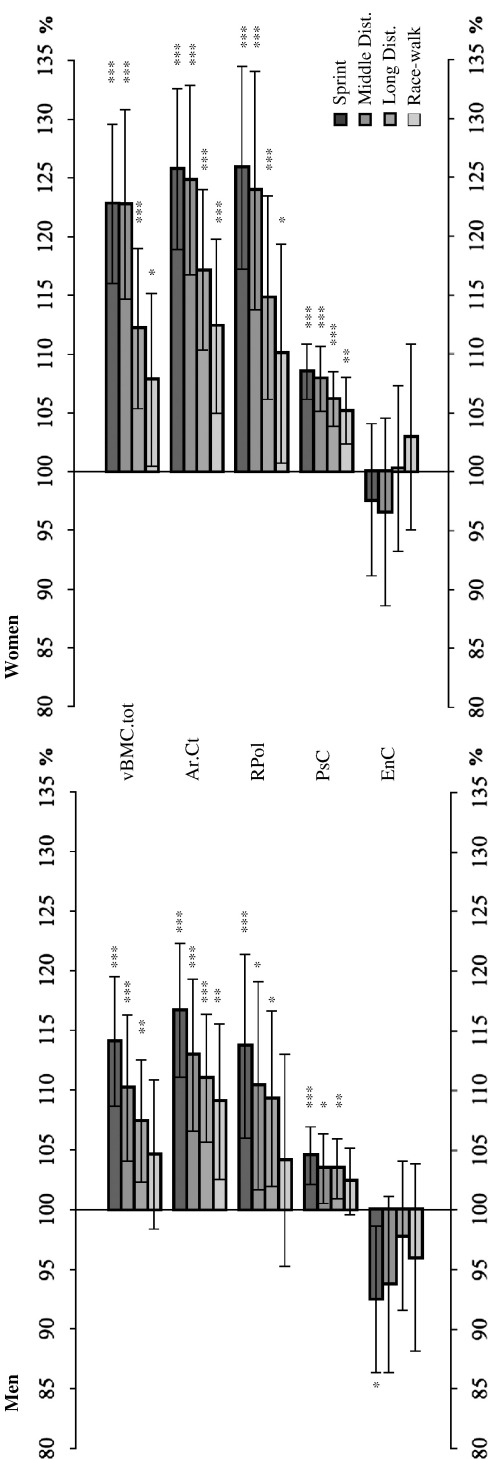
Percentage differences of bone measures of athletes and control participants at the tibia diaphysis. Bone measurement percentage differences of the athletes' tibia diaphysis compared to the control group (100%). Asterisks indicate a significant difference between the control group and the given athletic groups as follows: ⁎*p* < 0.05, ⁎⁎*p* < 0.01, ⁎⁎⁎*p* < 0.001. See Table 5 and Materials and methods for abbreviations.

Table 5 summarises the pQCT data from the tibia, and Fig. 1 compares each athlete group with the sedentary control people. As can be seen from the figure, diaphyseal vBMC.tot, RPol and Ar.Ct values were at least 13% and 23% greater in male and female sprinters compared to the control participants (*p* < 0.001). Similarly, diaphyseal PsC was also significantly larger in the male and female sprinters by 4% and 8%, respectively (*p* < 0.001). These measures were observed to decrease systematically as the speed declined, so that the race-walkers were least different compared to the controls (*p* < 0.05). Likewise, diaphyseal circularity was greater in all athlete groups compared to the control group (*p* < 0.001 for all female groups and male race-walkers; *p* < 0.01 or < 0.05 for all others). Conversely, diaphyseal vBMD.ct, as a measure closely linked to material properties, was 1–3% and 2–4% lower in male and female athletes, respectively, compared to the controls and showed a pattern that was distinct from that observed for the geometrical measures (*p* < 0.05; except for female middle distance runners, where *p* > 0.05).

Epiphyseal group differences were found particularly for bone mineral content and predominantly in the sprinters. Their vBMC.tb values were 15% and 18% larger in males and females, respectively, compared to control participants (*p* < 0.001).

Sex differences were observed for all tibial bone measures (*p* < 0.001), except for cortical vBMD and circularity (*p* > 0.4). Including the respective radius measure as a covariate, and thus accounting for ‘skeletal constitution’ (see Statistical analysis above), yielded either similar or greater significance levels for all diaphyseal tibia measures, and similar significance for all epiphyseal measures.

### Radius pQCT data

**Fig. 2:**
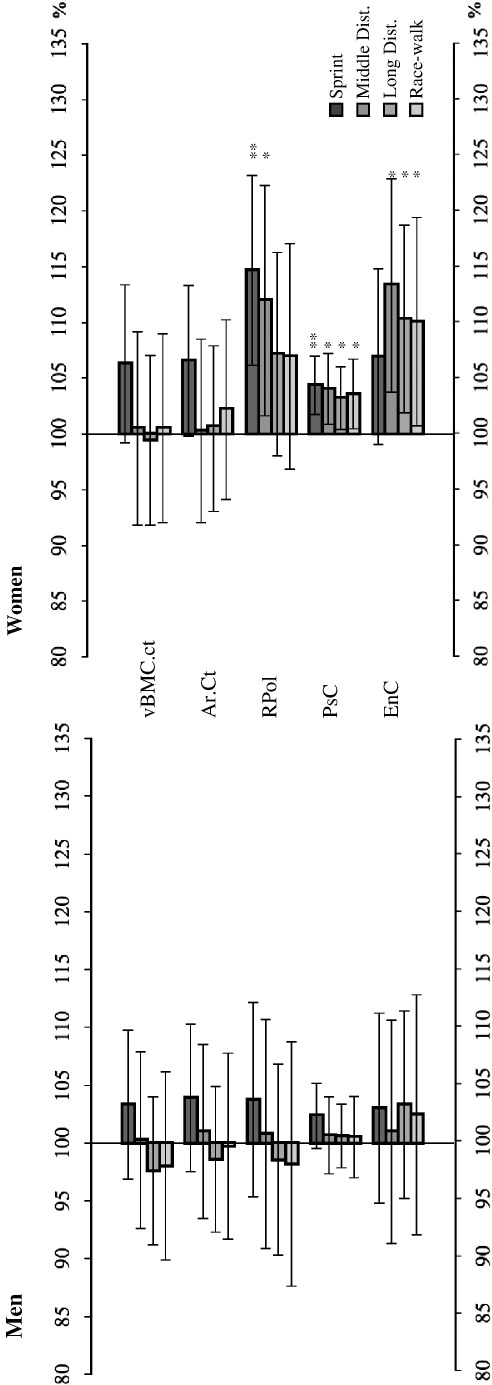
Percentage differences of bone measures of athletes and control participants at the radius diaphysis. Bone measurement percentage differences of the athletes' radius diaphysis compared to the control group (100%). Asterisks indicate a significant difference between the control group and the given athletic groups as follows: ⁎*p* < 0.05, ⁎⁎*p* < 0.01. See Table 6 and Materials and methods for abbreviations.

Bone scans from the non-weight bearing radius were obtained as ‘internal control’, in order to assess any potential selection bias (Table 6). Female middle distance runners and sprinters had 12% and 15% larger RPol values than control participants (*p* < 0.05) and all female athletes except for sprinters had 6–13% greater endocortical circumferences than the controls (*p* < 0.05), whilst male athletes did not differ in any of those measures to the controls (Table 6; Fig. 2). Cortical vBMD was approximately 1.5% smaller in both male and female long distance runners and race-walkers compared to the control group (*p* < 0.05). Moreover, female race-walkers had slightly larger epiphyseal vBMC.tb and vBMD.tb values than control participants, and in males group differences were observed between long distance runners and control participants for both Ar.tot and vBMD.tb (*p* < 0.05). Sex differences were significant for all bone measures (*p* < 0.001). In summary, the data reveal some athlete *vs.* control group differences, which were considerably smaller than those observed in the tibia for the respective bone measures.

## Discussion

This study has compared bone mass and geometrical measures of the weight-bearing tibia and the non-weight bearing radius of master sprinters, middle and long distance runners, race-walkers and sedentary control participants. As hypothesised, the greatest tibial surrogates of bone strength were found in sprinters and the lowest ones in control participants, except for cortical vBMD values for which a somewhat reverse pattern was observed. The non-weight bearing radius, which is not particularly loaded during running and walking, did nevertheless show some significant group effects.

### Tibia diaphysis: geometrical measures

Fig. 1 shows that athlete *vs.* control group differences were greatest for sprinters for all geometrical measures except for endocortical circumference. Also, and very clearly so, qualitatively similar observations were made in all other athlete groups, but the size of differences compared to the control participants became smaller as the discipline-specific speed declined. This systematic pattern, which is clearly present in all variables depicted in Fig. 1, is in apparent relation to the discipline-specific speeds. It is well understood that vertical ground reaction forces and tibia shaft strains, generated during running, increase as a function of running speed, and forces and strains peak at the same phase of the gait [13,20]. It therefore seems plausible to assume a causal relationship between running speed and greater mechanical competence of the tibia shaft. The nature of this relationship, however, cannot be determined from this study. On the one hand, the effectiveness of mechanical strain [21] and strain rate [22] on bone are well documented and have been conceptualised, *e.g.* in the ‘mechanostat’ theory [2], to explain the structural adaptation of bones to changing mechanical demands. There is therefore good reason to believe that discipline-specific musculoskeletal forces could have caused the group differences, and this notion is supported by slightly enhanced significance levels for athlete *vs.* control group differences after ‘adjustment’ of tibial with the respective radial pQCT measures, which may suggest that skeletal constitution confounds, rather than explains those group differences. On the other hand, it would be equally plausible to assume that larger bones offer the opportunity for larger muscles to attach and generate and transfer larger musculoskeletal forces, and that, consequently, larger bones may constitute a prerequisite for faster running. Either way, what this study suggests is that the tibia shaft is mechanically adapted to different running speeds.

The observation that athletic participation in high or odd impact sports was greater prior to puberty in athletes than in control people may be of quite some importance in the interpretation of our data, as there is accumulating evidence to suggest that the pre- and peri-pubertal phases constitute ‘windows of opportunity’ to enhance bone strength [23–27]. It is unclear, however, in how far these benefits can be transferred to adulthood [28], but it is possible that they elevate a postulated ‘upper threshold’ for bone strength during adulthood [29].

In our study, it is also of interest to quantitatively compare the different aspects of mechanical competence observed in the athletes' tibias. The polar moment of resistance, also called the section modulus, is a measure of structural engineering [30] and can serve as a surrogate of bone strength in torsion and also in bending [31,32]. In this study, RPol has been found to be 14% and 26% larger in the tibia shaft of male and female sprinters, respectively, compared to the control participants. Cortical area, or, since cortical bone mineral density shows very little inter-individual variation, also bone mineral content, can serve as a surrogate measure of bone strength in compression [18,33]. Quite interestingly, the group differences are very similar for diaphyseal RPol and vBMC.tot, the latter being 14% larger in male and 23% in female sprinters than in control people, and thus suggesting similar benefits for bending and compressive tibia strength for the athletes.

As expected, males had greater values for geometrical measures than females, but there was no interaction with age or the athletic group effects (Table 5). This is suggesting that, whatever the mechanisms behind the group differences, they will probably be the same for males and females.

### Tibia diaphysis: cortical vBMD

The differences in cortical vBMD between athletes and control participants were small, amounting to no more than 4%, but they were highly significant from a statistical point of view (Table 5). In keeping with the findings of Haapasalo et al. [34] that tennis players have reduced cortical vBMD in their dominant humerus shaft, athletes had generally lower cortical vBMD values than control people (exception: female middle distance runners). In the males, and to a lesser extent also in the females, cortical vBMD values appear to decrease in a very systematic way with running distance and training volume, so that endurance runners and race-walkers are most different from control people. It therefore seems that athlete *vs.* control group differences for cortical vBMD are somehow opposed to those observed for the other variables, *e.g.* BMC. It is well known that running induces micro-damage in bone [35], and that targeted remodelling helps to repair it [36]. Enhanced intracortical remodelling will lead to lower cortical vBMD values as assessed by pQCT. It would therefore be possible to explain the reduced cortical vBMD values in distance runners by the large number of deformation cycles their bones undergo during training and competition. This explanation would be in line with the observation by Schaffler and Burr that midshaft osteonal density was increased with mechanical usage in a comparison of 20 different primate species [37].

### Tibia epiphysis

It must be considered here that the distal tibia epiphysis is mainly composed of trabecular bone, and that mechanical strength of trabecular bone increases with trabecular density in a non-linear way. The latter relationship is best described by a power function, with an exponent of 2 or slightly greater [38,39]. Hence a difference in trabecular density by + 8%, as observed between the male sprinters and control people (Table 5) will ensue an increase in strength by 1.08^2^ = 1.17-fold, or by 17% — which is not far off the 15% that were actually observed for the difference in diaphyseal vBMC.tot between those groups. For the female sprinters, the corresponding values are + 29% for epiphyseal strength and + 25% for diaphyseal vBMC.tot, which again is in good agreement with our expectations. Our data therefore suggest that epiphyseal and diaphyseal bone strengths were equally enhanced in the athletes' tibias.

### Radius

Bone scans of the radius were obtained in this study with the idea to have an ‘internal’ control, as it has been described formerly that the non-weight bearing radius is comparable between athletes and control people [40]. Although we observed some tendency of especially power athletes having greater bone strength indicators, particularly in the diaphysis (Table 6; Fig. 2), group differences were mostly not significant. Greater bone strength measures in the radius of power athletes might be explained by their participation in upper-body weight training [10]. Moreover, garden work and upper-body physical exercise were exclusion criteria for the control group, but not for the athlete groups, which warrants caution in the overall interpretation of the radius data. On the other hand, long distance runners and race-walkers had smaller radius epiphyseal vBMD.tb and vBMC.tb values than control participants, and all female athlete groups except for sprinters had larger endocortical circumferences than the control women (Table 6; Fig. 2), all suggesting reduced mechanical competence of the radius in these groups. It is therefore fair to say that mechanical competence was systematically superior in all athlete groups as compared to the control group in the tibia, but not in the radius.

### Limitations

As discussed above, self-selection will have a role in this cross-sectional study. It can therefore not be decided on basis of these data alone whether athlete *vs.* control group differences emerged as a result of the tibia's adaptation to exercise-specific forces. Alternatively, people with a ‘better’ skeletal make-up, which might involve larger bones, may be more inclined to engage in athletics. As a further caveat of this study, most athletes train for and compete in more than one event, leading to some overlap between athletic groups. Moreover, some kinds of training, *e.g.* plyometric jumps may have had an effect upon the bone measurements and affected group differences. However, it should be considered that group overlap leads to an under-, rather than to an over-estimation of the true effects.

### Conclusion

In conclusion, we found bone mechanical competence to be larger in the tibia of athletes than of control participants in both males and females. Surrogate measures of bone strength increased, particularly in the tibia diaphysis, as the discipline-specific speed increased. The only exception to this pattern was cortical vBMD in the tibia, which appeared to decrease systematically with running distance or training volume. Our results are therefore consistent with the view that geometrical tibial measures are positively related to running speed and possibly the musculoskeletal forces arising from it, whilst cortical vBMD is negatively related to the frequency of loading.

## Figures and Tables

**Table 1:** Participant characteristics.

		Controls	Race-walk	Long distance	Middle distance	Sprint
Males	*N*	32	21	58	27	51
	Age [years]	54 (13)	57 (11)	62 (12)	58 (13)	59 (15)
	Height [cm]	177 (7)	173 (5)	173 (7)	175 (9)	174 (5)
	Body mass [kg]	81 (14)	70 (7)⁎⁎	66 (6)⁎⁎⁎	71 (12)⁎⁎	74 (8)⁎
	AGP [%]		77 (7)	77 (10)	83 (9)	88 (9)
Females	*N*	43	28	35	25	55
	Age [years]	59 (13)	54 (9)	59 (11)	55 (12)	59 (14)
	Height [cm]	160 (6)	162 (7)	164 (6)	162 (7)	163 (7)
	Body mass [kg]	67 (13)	59 (7)⁎⁎	56 (6)⁎⁎⁎	56 (6)⁎⁎⁎	59 (7)⁎⁎⁎
	AGP [%]		74 (10)	80 (10)	88 (6)	89 (7)

Age, anthropometric characteristics and age-graded performance (AGP), *i.e.* the performance in relation to the age- and sex-specific world record, are given as group means (SD in brackets). Asterisks denote significant differences from the control group, with: ⁎⁎⁎*p* < 0.001, ⁎⁎*p* < 0.01, ⁎*p* < 0.05. All other variables showed no significant group differences.

**Table 2:** Sport during young adulthood.

		Controls	Race-walk	Long distance	Middle distance	Sprint
Males	Local/national	3	12	34	18	34
		9%	57%	59%	67%	67%
	International	0	1	2	4	0
		0%	5%	3%	15%	0%
Females	Local/national	4	8	12	10	26
		9%	29%	34%	40%	47%
	International	0	1	1	2	6
		0%	4%	3%	8%	11%

Given is the number of participants who participated in organised sports during young adulthood and competed at either local to national level or at international level.

**Table 3:** Type of sport during puberty and young adulthood.

	Race-walk	Long distance	Middle distance	Sprint
Race-walk	0/1	0/0	0/0	0/0
	0%/2%	0%/0%	0%/0%	0%/0%
Long distance	0/1	0/3	0/2	1/3
	0%/2%	0%/3%	0%/4%	1%/3%
Middle distance	0/4	0/4	3/9	0/3
	0%/8%	0%/3%	6%/17%	0%/3%
Sprint	2/1	3/2	1/1	9/29
	4%/2%	3%/2%	2%/2%	8%/27%
High/odd impact	7/9	19/22	11/8	20/19
	14%/18%	20%/24%	21%/15%	19%/18%

Given is the number of participants who participated in organised sports before puberty/during young adulthood. High or odd impact types of sport were defined as proposed by Nikander et al. [5]. Data have been pooled for males and females.

**Table 4:** Persistence of current training regime.

	Race-walk	Long distance	Middle distance	Sprint
Males	18 (15)	24 (16)	17 (12)	18 (18)
Females	11 (12)	21 (14)	17 (11)	16 (12)

Given is the number of years that participants have been maintaining the current training regime in terms of training volume and type of training.

**Table 5:** pQCT results for the tibia.

	Epiphysis			Diaphysis				
	vBMC.tb	Ar.tot	vBMD.tb	vBMC.tot	Ar.Ct	RPol	vBMD.ct	circ
	[mg/mm]	[mm^2^]	[mg/cm^3^]	[mg/mm]	[mm^2^]	[mm^3^]	[mg/cm^3^]	
Males								
Control	151 (30)	1310 (200)	258 (35)	413 (44)	333 (37)	1982 (326)	1236 (27)	0.621 (0.022)
Race-walk	154 (25)	1340 (133)	255 (34)	432 (54)⁎	363 (43)⁎⁎	2064 (333)⁎⁎	1199 (24)⁎⁎⁎	0.651 (0.024)⁎⁎⁎
Long distance	157 (24)	1395 (161)⁎	252 (39)	443 (43)⁎⁎	370 (36)⁎⁎⁎	2166 (294)⁎⁎	1203 (32)⁎⁎⁎	0.640 (0.028)⁎⁎
Middle distance	162 (25)	1362 (143)	265 (37)	455 (38)⁎⁎⁎	376 (31)⁎⁎⁎	2188 (283)⁎⁎⁎	1216 (33)⁎	0.635 (0.033)⁎
Sprint	174 (25)⁎⁎⁎	1389 (157)⁎	279 (39)⁎	471 (51)⁎⁎⁎	388 (43)⁎⁎⁎	2254 (344)⁎⁎⁎	1220 (37)⁎	0.639 (0.027)⁎⁎

Females								
Control	109 (23)	1084 (124)	225 (44)	302 (38)	243 (31)	1295 (182)	1249 (38)	0.608 (0.025)
Race-walk	109 (19)	1127 (131)	215 (28)	325 (39)⁎	273 (33)⁎⁎	1426 (214)⁎	1201 (40)⁎⁎⁎	0.649 (0.033)⁎⁎⁎
Long distance	114 (23)	1128 (157)	225 (40)	338 (44)⁎⁎⁎	285 (35)⁎⁎⁎	1500 (276)⁎⁎⁎	1197 (43)⁎⁎⁎	0.647 (0.039)⁎⁎⁎
Middle distance	119 (16)	1093 (103)	244 (33)⁎	370 (49)⁎⁎⁎	303 (38)⁎⁎⁎	1605 (234)⁎⁎⁎	1232 (37)	0.634 (0.032)⁎⁎⁎
Sprint	129 (24)⁎⁎⁎	1121 (117)	256 (40)⁎⁎⁎	370 (50)⁎⁎⁎	305 (40)⁎⁎⁎	1629 (274)⁎⁎⁎	1219 (40)⁎⁎⁎	0.635 (0.028)⁎⁎⁎
*p*(♀ ≠ ♂)	< 0.001	< 0.001	< 0.001	< 0.001	< 0.001	< 0.001	0.41	0.49

Mean values and standard deviations (SD) for bone mineral content (vBMC.tb), epiphyseal total bone area (Ar.tot), epiphyseal trabecular bone mineral density (vBMD.tb), diaphyseal cortical area (Ar.Ct), cross-sectional polar moment of resistance (RPol), cortical bone mineral density (vBMD.ct) and circularity (circ). *p*-values for sex differences are given in the bottom row. Asterisks indicate significant absolute differences from the control group: ⁎*p* < 0.05, ⁎⁎*p* < 0.01, ⁎⁎⁎*p* < 0.001.

**Table 6:** pQCT results for the radius.

	Epiphysis			Diaphysis			
	vBMC.tb	Ar.Ttavg	vBMD.tb	vBMC.tot	Ar.Ct	RPol	vBMD.ct
	[mg/mm]	[mm^2^]	[mg/cm^3^]	[mg/mm]	[mm^2^]	[mm^3^]	[mg/cm^3^]
Males							
Controls	44.8 (11)	440 (78)	227 (44)	127 (18)	127 (18)	364 (76)	1251 (30)
Race-walk	41.2 (8.1)	443 (61)	207 (31)	124 (17)	124 (17)	358 (68)	1231 (36)⁎
Long distance	42.8 (8.7)	474 (59)⁎	202 (39)⁎⁎	124 (18)	124 (18)	359 (69)	1236 (27)⁎
Middle distance	45.1 (7.5)	457 (58)	221 (34)	127 (16)	127 (16)	367 (67)	1243 (33)
Sprint	46.2 (10)	467 (58)	221 (42)	131 (19)	131 (19)	378 (68)	1242 (28)

Females							
Controls	28.7 (5.6)	352 (52)	183 (37)	92.7 (13)	75.7 (9.9)	213 (44)	1264 (32)
Race-walk	24.9 (5.5)⁎	360 (74)	158 (39)⁎⁎	93.2 (14)	77.3 (12)	228 (48)	1245 (31)⁎
Long distance	28.1 (8.0)	363 (59)	172 (42)	92.2 (15)	76.1 (12)	229 (44)	1244 (34)⁎⁎
Middle distance	29.8 (8.0)	361 (53)	184 (44)	93.2 (21)	75.9 (16)	239 (58)	1264 (37)
Sprint	30.6 (7.9)	367 (49)	186 (43)	98.5 (17)	80.6 (13)	245 (49)⁎⁎	1258 (33)
*p*(♀ ≠ ♂)	< 0.001	< 0.001	< 0.001	< 0.001	< 0.001	< 0.001	< 0.001

Mean values and standard deviations (SD) for bone mineral content (vBMC.tb), epiphyseal total bone areas (Ar.tot), epiphyseal trabecular bone mineral density (vBMD.tb), diaphyseal cortical area (Ar.Ct), cross-sectional polar moment of resistance (RPol), cortical bone mineral density (vBMD.ct) and circularity (circ). *p*-values for sex differences are given in the bottom row. Asterisks indicate absolute significant differences from the control group: ⁎*p* < 0.05, ⁎⁎*p* < 0.01.
